# Efficient and Low-Complex Signal Detection with Iterative Feedback in Wireless MIMO-OFDM Systems

**DOI:** 10.3390/s23249798

**Published:** 2023-12-13

**Authors:** Ying Chen, Yue Tang, Bin Jiang, Yinan Zhao, Jianrong Bao, Xianghong Tang

**Affiliations:** 1 Information Engineering College, Hangzhou Dianzi University, Hangzhou 311305, China; cheny@hdu.edu.cn; 2 School of Communication Engineering, Hangzhou Dianzi University, Hangzhou 310018, China; tx@hdu.edu.cn (Y.T.); jiangbin@hdu.edu.cn (B.J.); baojr@hdu.edu.cn (J.B.); tangxh@hdu.edu.cn (X.T.); 3 Sichuan Provincial Energy Investment Group Co., Ltd., Chengdu 610041, China; 4 National Mobile Communications Research Laboratory, Southeast University, Nanjing 210096, China

**Keywords:** MIMO-OFDM, iterative feedback, optimal detection, matrix inversion, computational complexity simplification

## Abstract

To solve error propagation and exorbitant computational complexity of signal detection in wireless multiple-input multiple-output-orthogonal frequency division multiplexing (MIMO-OFDM) systems, a low-complex and efficient signal detection with iterative feedback is proposed via a constellation point feedback optimization of minimum mean square error-ordered successive interference cancellation (MMSE-OSIC) to approach the optimal detection. The candidate vectors are formed by selecting the candidate constellation points. Additionally, the vector most approaching received signals is chosen by the maximum likelihood (ML) criterion in formed candidate vectors to reduce the error propagation by previous erroneous decision, thus improving the detection performance. Under a large number of matrix inversion operations in the above iterative MMSE process, effective and fast signal detection is hard to be achieved. Then, a symmetric successive relaxation iterative algorithm is proposed to avoid the complex matrix inversion calculation process. The relaxation factor and initial iteration value are reasonably configured with low computational complexity to achieve good detection close to that of the MMSE with fewer iterations. Simultaneously, the error diffusion and complexity accumulation caused by the successive detection of the subsequent OSIC scheme are also improved. In addition, a method via a parallel coarse and fine detection deals with several layers to both reduce iterations and improve performance. Therefore, the proposed scheme significantly promotes the MIMO-OFDM performance and thus plays an irreplaceable role in the future sixth generation (6G) mobile communications and wireless sensor networks, and so on.

## 1. Introduction

Multiple-input multiple-output (MIMO) was combined with orthogonal frequency division multiplexing (OFDM) to both effectively improve the spectrum utilization of communication systems and resist the channel interference and noises [1,2]. The multiple fading effect was utilized as a new signal reception quantity to be accumulated rather than the multipath fading distortion in MIMO-OFDM systems, which significantly increased the link capacity. In addition, the efficient and low-complexity signal detection was an important guarantee for a large-scale MIMO-OFDM system to be better used in the future 6G networks. There were two signal detection methods to be applied in MIMO-OFDM systems, including linear and nonlinear detection [3,4]. Additionally, three deep neural network (DNN) models, i.e., the DNN model to de-map the signals, the DNN model to learn and filter out the channel noises, and the DNN model to de-map and detect the signals, were suggested to solve non-linear distortions caused by the power amplifier of the transmitters and replace the conventional digital signal processing modules at the receivers [5]. In [6], the zero forcing (ZF) and minimum mean square error (MMSE) algorithms with linear detection were compared and analyzed. The detection performance of the former was much lower than that of the latter due to excessive amplification of noises. But, there was still a big gap between the MMSE and maximum likelihood (ML) algorithms. The detection and computational complexity of several commonly used nonlinear detection methods were studied in [7]. Differing from linear detection, the nonlinear one improved the detection performance to a great extent, but at the expense of increasing computational complexity. Among nonlinear detection, the ordered successive interference cancellation (OSIC) algorithm was more suitable for use in practice. To make up for the shortcomings of the above detection algorithms, the MMSE and OSIC methods were combined together to obtain an even better performance of both optimal MMSE and less complex OSIC detection [8]. On the basis of MMSE detection, nonlinear feedback was used to suppress and eliminate the interference from the strong to weak according to signal-to-noise ratios (SNRs) or signal-to-interference-plus-noise ratios (SINRs) of received signals in each transmitting antennas [9,10]. In this way, it further improved the detection performance to approach the optimum detection. In practice, the MIMO-OFDM transmissions become a widely accepted air interface to ensure a significant improvement in spectral efficiency. For the future massive MIMO-OFDM systems, a mathematical expression was introduced to enable the analysis of the energy detection performance with the square-law combining method [11]. Analyses were performed for detecting primary user (PU) signals by the secondary user (SU) exploiting the MIMO-OFDM. Comprehensive simulation analyses of energy detection was performed via the square-law combining (SLC) method for versatile combinations of operating parameter characteristics for different working environments of MIMO-OFDM systems [12]. In summary, the performance of nonlinear detection for MIMO-OFDM systems was better than that of the linear one. However, without considering computational complexity, the combination of them made the effect better. On this basis, they are further made up for defects of error propagation, which makes the detection performance of the system to be optimum.

Except that the detection performance can be further enhanced, the complexity improvement of the algorithm was another key issue to be optimized in wireless communication systems. In large-scale MIMO systems, with the number of antennas at the base station increasing dramatically, the channels were asymptotically orthogonal [13,14]. The complexity of the MMSE detection required cubic growth with the number of transmitting antennas due to the introduction of matrix inversion operations. To reduce the computational complexity from matrix inversion, signal detection algorithms based on Neumann series expansion was proposed in [15]. The inverse matrix was approximated via polynomial expansion. However, when the number of iterations increased gradually, the computational complexity was not significantly decreased. Similarly, another multistage linear receiver method was proposed in [16] to approximate the MMSE exact matrix inversion. The computational complexity reduction method was similar to the Neumann series expansion, but the difference was that this scheme achieved a better detection performance via low order extension. In addition to the idea of polynomial expansion, an algorithm called Richardson with an iterative method was proposed in [17]. It estimated the transmitted signal vector directly at the receivers and then converted the matrix inversion simplified problem to that of solving the linear equations. A symmetric successive over-relaxation (SSOR) algorithm was also proposed with an iterative method, which effectively avoided the complex matrix inversion process and thus significantly reduced the computational complexity of the entire system [18]. In this paper, the matrix inversion process of the MMSE algorithm is simplified approximately and the traditional OSIC algorithm is ameliorated via the multilayer merging detection. As a result, the proposed scheme both improves the detection performance and reduces the computational complexity significantly. Therefore, the system improves both the bit error ratio (BER) and detection efficiency.

Since there are many abbreviations used in this paper, the list of abbreviations is summarized and shown in the abbreviation section for clarity.

Finally, the effectiveness of the proposed scheme is tested and verified via numerical simulations with some analyses of the performance improvement and complexity reduction. The main contributions are summarized as follows.

- An iterative feedback MMSE-OSIC algorithm is proposed with a reliability constraint. By selecting the best candidate vector, the error propagation genetic problem in the previous decoding is improved. Compared with the traditional MMSE-OSIC algorithm, the proposed scheme improves the performance of about 4∼5 dB at the same BER.
- A modified SSOR iterative algorithm is proposed to avoid complex computation of matrix inversion. Given the quantified relaxation parameters and initial values, the SNR is improved about 5 dB at the same BER compared with that of the traditional method. Additionally, the convergence speed is accelerated too. With the number of iterations of 2∼3, the detection performance is just close to that of the MMSE method.
- An optimization strategy of the OSIC algorithm is proposed. On the condition of maintaining a relative satisfactory performance, *m* layers are selected according to the formula of computational complexity. In addition, the parallel detection and corresponding accurate detection value are adopted to achieve the goal of minimum computational complexity.

This paper is concisely introduced as follows. Section 2 presents an MMSE-OSIC signal detection model for MIMO-OFDM systems. Section 3 introduces a joint improvement of the MIMO-OFDM signal detection model for better performance and less complexity. Section 4 gives quantitative analyses of the overall computational complexity of the proposed signal detection scheme in MIMO-OFDM systems. Section 5 presents simulation results and performance analyses to verify the apparent good performance of the proposed scheme. Finally, Section 6 concludes the whole paper.

## 2. MMSE-OSIC Signal Detection Model for MIMO-OFDM Systems

The MIMO and OFDM are combined together to form the MIMO-OFDM system and it both obtains the effective spatial multiplexing gain and improves the data transmission rate and bandwidth utilization [19,20]. In addition, the frequency selective fading in MIMO systems is effectively tackled by taking advantage of the OFDM characteristics, so that the system is not easily interfered by inter-symbol interferences (ISIs) and inter-carrier interferences (ICIs) [21].

**Figure 1:**
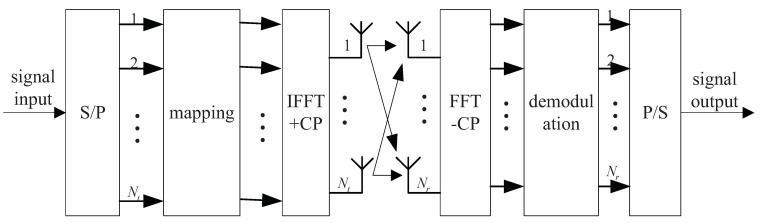
Block diagram of signal transmission model of the MIMO-OFDM system.

The block diagram of the MIMO-OFDM system transmission model is presented in Figure 1.

In the above system model, the number of transmitting antennas is $N_{t}$, the number of receiving antennas is $N_{r}$, and the number of the OFDM subcarriers is *k*. The mathematical model of the MIMO-OFDM system is expressed as

**Figure 2:**
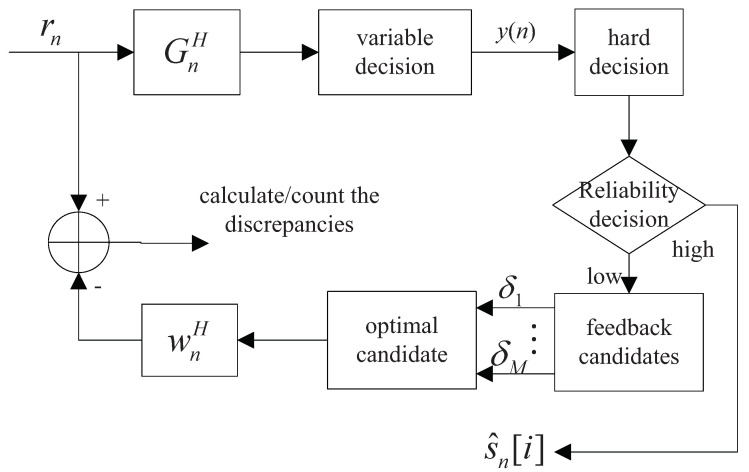
Block diagram of the MMSE-OSIC scheme with optimal constellation feedback.

(1) r(k)=H(k)s(k)(k)+n(k),

where s(k) is the vector of transmitted signals, n(k) is the vector of noises in transmissions, r(k) is the vector of receiving signals in the system, and H(k) is the channel matrix between the transmitting and receiving antennas. In addition, H(k) is further expressed as

(2) $$H\left(k\right)=\left[\begin{array}{cccc} h_{1,1}\left(k\right) & h_{1,2}\left(k\right) & \cdots & h_{1,N_{t}}\left(k\right)\\ h_{2,1}\left(k\right) & h_{2,2}\left(k\right) & \ldots & h_{2,N_{t}}\left(k\right)\\ \vdots & \vdots & \ddots & \vdots \\ h_{N_{r},1}\left(k\right) & h_{N_{r},2}\left(k\right) & \cdots & h_{N_{r},N_{t}}\left(k\right) \end{array}\right],$$

where $h_{j,i}\left(k\right)$ represents the channel gain and the information in transmitting antenna *i* is received by the receiving antenna *j*. Given that the received signals of the MIMO-OFDM system are acquired, it is necessary to find a signal detection criterion to obtain the estimated value of transmitted signals. The maximum likelihood (ML) scheme has the optimal detection performance, but the number of search paths is too large, resulting in huge computational complexity. Therefore, a constellation feedback optimization is proposed with the sub-optimal MMSE-OSIC algorithm. It effectively reduces the probability of error propagation caused by the previous erroneous decision. After the decision in the OSIC algorithm, a procedure of feedback operation based on the optimal constellation point is increased. The detection layers with low reliability after the soft estimation is then redetermined. Finally, the improved detection model is constructed and shown in Figure 2.

In Figure 2, the block containing $G_{n}^{H}$ represents the detection weight vector after the update channel matrix in the OSIC detection. Additionally, the operator ${\left(\cdot \right)}^{H}$ means the operation of conjugation and transposition. The block with $w_{n}^{H}$ represents each column of the updated filtered matrix in the OSIC algorithm. The block with ${\hat{S}}_{n}$ represents the output of detected signals after iterative feedback and decision. By using the orthogonal criterion and normalizing the power of the system, the filter matrix is expressed as

(3) $$G_{\mathrm{MMSE}\;}={\left(H^{H}H+N_{0}I_{N_{r}}\right)}^{-1}H^{H},$$

Thus, the filtered output is expressed as

(4) $${\hat{s}}_{MMSE}=G_{MMSE}r={\left(H^{H}H+N_{0}I_{N_{r}}\right)}^{-1}H^{H}\cdot r,$$

After the completion of the MMSE equalization, the signal-to-interference-plus-noise ratio (SINR) is used as a reliability measure to sort the signals in each layer, to effectively suppress the error propagation. The SINR $\rho_{i}^{j}$ corresponding to the transmitted signals in the *j*-th path of the *i*-th detection is given as

(5) $$\rho_{i}^{j}=\frac{G_{i}^{j}\cdot h_{i}^{j}\cdot \sigma_{s}^{2}}{\sigma_{n}^{2}\cdot G_{i}^{j}\cdot {\left(G_{i}^{j}\right)}^{*}+\sigma_{s}^{2}\cdot \sum_{p\neq k_{1},\cdots ,k_{i}}^{N_{t}}G_{i}^{j}\cdot h_{p}^{j}}$$

where $\sigma_{s}^{2}$ and $\sigma_{n}^{2}$ represent the emission energy of signals and noises, respectively. $G_{i}^{j}$ is the *i*-th element of the detection weight vector after the update channel matrix in the *j*-th path of (3). $h_{i}^{j}$ is the *i*-th element of channel coefficients in the *j*-th path of (3). $N_{t}$ represents the number of transmission antennas. ${\left(\cdot \right)}^{*}$ represents the conjugation operation. The SINR of each layer is closely related to the column vector of the MMSE filtering matrix.

## 3. Improved MIMO-OFDM Signal Detection for Both Good Performance and Low Complexity

### 3.1. Performance Optimization via Iterative Feedback Detection

In view of the error propagation phenomenon, an improved MMSE-OSIC detection on the basis of iterative feedback is proposed. After the decision process in the traditional OSIC algorithm, a step of feedback operation by employing the optimal constellation point is increased. The detection layers with low reliability after soft estimation are output after re-decision. The key steps of the optimal candidate vector and re-decision are as follows.

**Figure 3:**
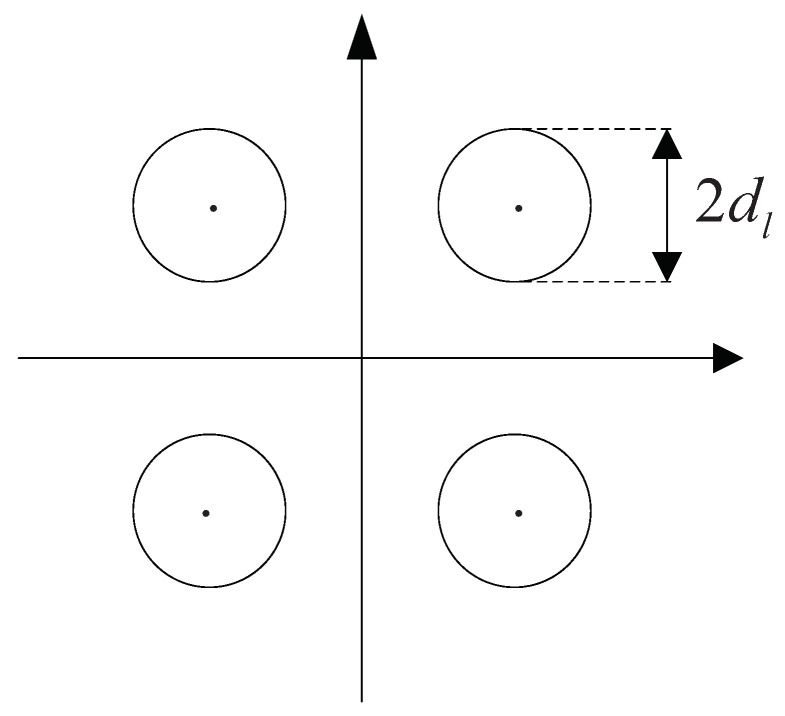
Constellation for the reliability determination of the QPSK.

- Selection of the optimal candidate vector
  The selection of the optimal feedback candidate point is the most important procedure in the proposed algorithm. When multiple phase-shift keying (MPSK) modulation is adopted, *M* candidate feedback constellation points $\left\{p_{1},p_{2},\ldots ,p_{M}\right\}$ are generated. The concrete structure and selection method are illustrated as follows.
  First, the difference with the decision and received signals is expressed as
  (6) $$c_{k}=\hat{r}-\sum_{l=k}^{Nt}H{\left(l\right)}^{*}\hat{s}\left(l\right)$$
  Then, *M* candidate vectors are constructed from other layers to be detected.
  (7) $$\begin{array}{c} \eta_{1}\to {\hat{s}}_{1},{\hat{s}}_{2},\ldots ,p_{1},c_{k+1},\ldots ,c_{Nt}\\ \eta_{2}\to {\hat{s}}_{1},{\hat{s}}_{2},\ldots ,p_{2},c_{k+1},\ldots ,c_{Nt}\\ \vdots \\ \eta_{M}\to {\hat{s}}_{1},{\hat{s}}_{2},\ldots ,p_{M},c_{k+1},\ldots ,c_{Nt} \end{array}$$
  The optimal candidate vector is selected from the formed *M* vector branches by using the ML criterion.
  (8) $$\eta =\arg\min∥r-H^{*}\lambda_{n}∥,1<n<M$$
- Reliability decision and output of detection signals
  The reliability of the estimated signals are mainly determined by the distance between soft estimation points and candidate constellation ones. The soft estimation result from Figure 2 is expressed as
  (9) $$y\left(k\right)=G_{k}^{H}*r_{k}$$
  The distance $d_{k}$ between the soft estimate point and its nearest constellation point is expressed as
  (10) $$d_{k}=\left|y\left(k\right)-\lambda_{c}\right|$$
  where $\lambda_{c}$ is the constellation point nearest to the soft estimation output of the *k*-th layer.
  After hard decision of the detected signals, the constellation point is introduced as the feedback candidate point. The hard decision output point coordinates are compared with the introduced constellation candidate point. Then, a reliable threshold of decision $d_{l}$ is given. The threshold is adjusted according to the specific conditions of the channel. Take the quadrature phase shift keying (QPSK) modulation as an example, of which the reliability constraint criterion after the threshold setting is shown in Figure 3.
  The centers of these four circles represent the candidate constellation points. On the one hand, when $d_{k}<d_{l}$, soft estimation points fall in the circular region and the judgment is reliable. At this time, the estimated signals are output directly. On the other hand, when $d_{k}>d_{l}$, it represents that the decision point is in the unreliable region. Additionally, all the points outside the circular region need to output after decision by taking advantage of the best constellation feedback point.
  For the undetected layer, the conventional OSIC algorithm is used to obtain the detection results directly. The candidate constellation points and detection results in subsequent layers are used to form candidate vectors. Finally, an optimal vector is selected from candidate vectors of the output as the test results. In this way, the estimated value of the transmitted signals are obtained by completing the above procedures for each layer. In addition, the channel matrix needs to be updated until all the detection is completed.

### 3.2. Low-Complexity Iterative Approximation Optimization for Matrix Inversion

The proposed detection achieves a good signal detection performance, but it also needs a traditional MMSE algorithm to complete the filtering process before detection. Since the MIMO system is usually a large antenna transceiver system, it is necessary to inverse the channel matrix in the filtering process. With the increase in the antennas, the calculation of matrix inversion is very huge. So, the approximation strategy using a quantitative relaxation factor with the modified SSOR algorithm is applied on such occasions for low implementation complexity. By means of the iterative calculation, the approximation strategy with the quantitative relaxation factor is efficiently used in each iteration without huge computation, and the fine solution can be obtained with only several iterations by choosing the proper initial quantitative relaxation factor with the successive procedures and related equations from (11) to (22). Therefore, effective complexity reduction in matrix inversion in signal detection is an important research content of MIMO-OFDM system transmission.

It is required to reduce the computational complexity, but it still ensures the detection performance of the system simultaneously. Then, the following measures are made based on the iteration to replace the exact matrix inversion process with approximate iterative results. To facilitate the explanation, define $W=H^{H}H+N_{0}I_{N_{r}}$ and $r_{d}=H^{H}r$, then (4) is expressed as

(11) $$\hat{s}=W^{-1}r_{d},$$

or

(12) $$W\hat{s}=r_{d}.$$

Thus, the complex matrix inversion process is simplified as the solution to the linear equation. W is a symmetric positive definite matrix. According to the structural characteristics of positive definite matrix, the matrix is decomposed in this chapter. The complexity optimization is processed by using the modified SSOR iterative algorithm [18]. To solve linear equations and explain the corresponding complexity analysis better, the matrix is decomposed via the simple matrix decomposition as

(13) W=D−L−U,

where D, L, and U are the diagonal matrix and strict lower and upper triangular matrix of W, respectively, and $L=U^{T}$. Since the MIMO system is usually a large antenna transceiver system, it is necessary to inverse the channel matrix in the filtering process. With the increase in the antennas, the calculation of matrix inversion is huge. So, the approximation strategy with the quantitative relaxation factor with the modified SSOR algorithm is applied on such occasions for low implementation complexity. By means of the iterative calculation, the approximation strategy with the quantitative relaxation factor is efficiently used in each iteration without huge computation, and the fine solution can be obtained with only several iterations by choosing the proper initial quantitative relaxation factor with the successive procedures. Then, the recursive estimation information of the transmitted signals is obtained via the above modified SSOR algorithm as

(14) $${\hat{s}}^{\left(t+1\right)}={\left(\omega D-L^{T}\right)}^{-1}\left[\left(\omega -1\right)L^{T}+\omega L\right]{\hat{s}}^{\left(t\right)}+{\left(D-\frac{1}{\omega }L^{T}\right)}^{-1}r_{d}.$$

In (14), the relaxation factor ω and the iterative initial value must be configured appropriately to obtain better estimation results. They are all necessary parameters in the above-modified SSOR algorithm to adjust the iteration process for low complexity and high resolution. The relaxation factor has an important influence on the convergence and convergence conditions. In other words, for an *N*-dimensional linear equation, the relaxation factor must be able to converge the equation to a real solution. Define the iterative matrix as

(15) $$F={\left(\omega D-L^{T}\right)}^{-1}\left(\left(\omega -1\right)L^{T}+\omega L\right).$$

To obtain a convergence of (14), the spectral radius of the iterative matrix needs to be satisfied with

(16) $$\varphi \left(F\right)=\max_{1\leq n\leq 2N_{r}}\left|\lambda_{n}\right|<1$$

where $\lambda_{n}$ represents the *n*-th eigenvalue of F, and it satisfies the following equations.

(17) $$\lambda_{n}^{2}=\frac{{\left(2\omega {\hat{s}}^{T}L\hat{s}-{\hat{s}}^{T}L\hat{s}\right)}^{2}}{{\left(\omega {\hat{s}}^{T}D\hat{s}-{\hat{s}}^{T}L\hat{s}\right)}^{2}}$$

When the $N_{t}$ and $N_{r}$ are large enough, the maximum eigenvalue of the matrix W in (13) is approximately expressed as

(18) $$\lambda_{\max}=N_{t}{\left(1+\sqrt{N_{r}/N_{t}}\right)}^{2}$$

Subsequently, a near optimal relaxation factor $\omega_{s}$ after quantization is obtained, which is mainly determined by the system parameters $N_{t}$ and $N_{r}$.

(19) $$\omega_{s}=\frac{2}{\left(1+\sqrt{2\left(2-{\left(1+\sqrt{N_{r}/N_{t}}\right)}^{2}\right)}\right)}$$

In addition, to facilitate the implementation of the algorithm and simplify the calculation, the initial value is usually set to the zero vector of the 2$N_{r}$× 1 dimension in the traditional relaxation iterative algorithm. The initial value setting of the iterative algorithm does not affect the convergence of the algorithm, but it affects the convergence speed of the algorithm to some extent. Therefore, to further improve the convergence speed and reduce the computational complexity, a more reasonable iterative initial value is proposed via the channel hardening in large-scale MIMO systems. With the increase in row and column numbers of the channel matrix H, compared with those of the diagonal elements, the value of the non-diagonal elements $H^{H}H$ become smaller. By the channel hardening, the approximate value of $H^{H}H$ is obtained as

(20) $$H^{H}\cdot H\approx N\cdot I_{2N_{r}}.$$

Simultaneously, the inversion result of matrix **W** is presented as

(21) $$W^{-1}\approx D^{-1}\approx {\left(N_{t}+\sigma^{2}/E_{s}\right)}^{-1}I_{2K}\approx N_{t}^{-1}I_{2K}.$$

Therefore, the iterative initial value of (14) is set as

(22) $${\hat{s}}^{\left(0\right)}=\frac{1}{N}I_{2N_{r}}\cdot r_{d}.$$

After the improvement of the above algorithm, the computational complexity is reduced from O($N_{r}^{3}$) to O($N_{r}^{2}$).

### 3.3. Multilayer Merging in Joint Detection Strategy for the OSIC Algorithm

In this chapter, to maintain a relatively satisfactory signal detection performance, the number of pseudo inverse operations in the detection algorithm is reduced as much as possible. According to the steps of the OSIC algorithm, it determines the detection sequence via the SINR of signals. The detection effect of the first few layers significantly affects the detection results of the subsequent detection layers and even the error performance of the entire MIMO-OFDM system.

On the basis of the detection method layer by layer, the multilevel joint parallel detection strategy is used to optimize the detection processes of the system. An improved detection algorithm is also proposed via the derivation of the complexity equation, which makes it a good tradeoff between performance and complexity. If the small performance loss caused by parallel detection can be tolerated, the improved algorithm significantly reduces the computational complexity. First, according to the complexity formula of the algorithm, *m* layers with better SINR are selected as the improved detection target to complete the rough multilevel merging estimation and also the judgment and decision. The detection results are then listed as

(23) $${\left[y_{1},y_{2},\ldots ,y_{m}\right]}^{T}=Q\left({\left[{\left(w_{1}\right)}^{T},{\left(w_{2}\right)}^{T},\ldots ,{\left(w_{m}\right)}^{T}\right]}^{T}r\right).$$

To make the detection much more accurate, the interference between the selected *m* layers are dispelled by using the elimination of the front and back items.

(24) $$r_{j}=r-\sum_{i=0}^{j-1}{\hat{s}}_{i+1}{\left(H\right)}_{i+1}-\sum_{i=j+1}^{m-1}y_{i+1}{\left(H\right)}_{i+1}.$$

Finally, the detected output signals are acquired via the judgment on the results of the joint detection information.

(25) $${\hat{x}}_{1+j}=Q\left(\left[W_{1+j}\right]r_{j}\right)$$

In this way, the (m−1) recursions in the OSIC algorithm are eliminated. Thus, (m−1) matrix pseudo inverse operations are saved.

### 3.4. The Entire Procedure for the MIMO-OSIC Scheme

Based on the analyses of the above MIMO and OSIC algorithms, especially the basic block diagram of the MMSE-OSIC scheme with optimal constellation feedback, the entire procedures for the MIMO-OSIC scheme are summarized and presented as follows.

- The matrix $G_{n}^{H}$ is calculated by (3) with the MMSE criterion and it represents the detection weight vector after the update channel matrix in the OSIC detection.
- The input variable $r_{n}$ passes through the block with $G_{n}^{H}$ and performs the calculation of (4) to obtain the output variable ${\hat{s}}_{MMSE}$. The decision variable y(n) is obtained by (9).
- After the MMSE equalization, the SINR is used as a reliability measure to sort the signals in each layer, to effectively suppress the error propagation. The SINR $\rho_{i}^{j}$ corresponding to the transmitted signals in the *j*-th path of the *i*-th detection is shown in (5).
- The decision variable y(n) is firstly performed with hard decision and the result is sent and judged in the diamond block of reliability.
- The detection layers with low reliability after soft estimation are output after re-decision. The key steps of the optimal candidate vector and re-decision in the reliability decision are as follows.
  - Selection of the optimal candidate vector: The selection of the optimal feedback candidate is the most important procedure in the proposed algorithm. Given MPSK modulation, *M* candidate feedback constellation points $\left\{p_{1},p_{2},\ldots ,p_{M}\right\}$ are generated. The concrete structure and selection method are illustrated as in (6) and (7). Then, the optimal candidate vector is selected from the formed *M* vector branches by using the ML criterion in (8).
  - Reliability decision and output of detection signals: The reliability of the estimated signals are mainly determined by the distance between soft estimation points and candidate constellation ones. The soft estimation result is expressed in (9). The distance $d_{k}$ between the soft estimate point and its nearest constellation point is expressed as in (10) with the corresponding variables defined around it. After hard decision of the detected signals, the constellation point is introduced as the feedback candidate point. The hard decision output point coordinates are compared with the introduced constellation candidate point. Then, a reliable threshold of decision $d_{l}$ is given. The threshold is adjusted according to the specific conditions of the channel. Finally, the reliability constraint criterion after the threshold setting is shown in Figure 3 and the reliability decision is made just as it.
- The centers of these circles represent the candidate constellation. Given $d_{k}<d_{l}$, soft estimation points fall in the circular region and the judgment is reliable. The estimated signals are output directly. Otherwise, given $d_{k}>d_{l}$, the decision point is in the unreliable region. All the points outside the circular region need to output after decision via the best constellation feedback point.
  For the undetected layer, the OSIC is used to obtain the detection results directly. The candidate constellation points and detection results in subsequent layers are used to form candidate vectors. Finally, an optimal vector is selected from candidate vectors of the output as the test results. By this mean, the estimated value of the transmitted signals are obtained by completing the above procedures for each layer. In addition, the channel matrix needs to be updated until all detection is completed. In addition, some computation techniques, such as low-complexity iterative approximation optimization for matrix inversion, is adopted for low complexity, and they will be discussed later.

## 4. Quantitative Analyses of the Overall Complexity of the MIMO-OFDM System

Compared with the traditional MMSE-OSIC, the proposed MMSE-OSIC with feedback significantly improves the detection performance, but the computational complexity is also increased due to the introduction of feedback process, which mainly includes the following issues.

- When the soft decision point is in the shadow region, it is necessary to calculate the distance from the soft decision point to the horizontal ordinate coordinate, and *M* more additions are required.
- *M* nearest constellation points are selected as candidates. Additionally, each layer needs to add *M* additions.
- The soft decision from (l+1)-th to the $N_{t}$-th layers are computed, and then *M* candidate column vectors are obtained. This process requires $M\times \left(N_{t}-l\right)\times \left(2N_{t}-1\right)$ additions and $M\times N_{t}^{2}$ multiplications.
- The optimal candidate is selected by Formula (8). $M\times N_{t}^{2}$ additions and $M\times N_{t}^{2}$ multiplications are needed. Thus, the detection of the *l*-th layer demands to be increased with $\left[P\times 3M\times N_{t}^{2}-M\times \left(l+1\right)\times N_{t}+M\times l+2M-1\right]$ additions and $P\times N_{t}\left(3M\times N_{t}-2M\times l+1\right)$ multiplications, respectively.
  The computational complexity of the MMSE algorithm is $O(N_{r}^{3})$, mainly derived as M. When the matrix inversion process is replaced approximately by using (14), the computational complexity of each iteration decreases to $O(N_{r}^{2})$. For each iteration, the computational complexity remains unchanged. Although the OSIC algorithm significantly reduces the computational complexity compared with that of the ML algorithm, it still needs times of the pseudo inverse matrix operation. Moreover, it also need operations to complete the sorting and interference cancellation. Suppose that *m* layers are selected to finish parallel cancellation, the computational complexity of the improved algorithm is
  (26) $$\begin{array}{cc} C= & \left(4N_{r}{}^{3}+2N_{t}N_{r}{}^{2}\right)+3N_{t}m+2\left(N_{t}-1\right)m+\left(m-1\right)m\\ \; & +\left(13N_{r}{}^{3}+N_{r}{}^{2}\right)\left(N-m\right) \end{array}.$$
  Thus, there exists an *m* that minimizes computational complexity *C* of the scheme. To select the optimal layer number, the calculation complexity of several detection algorithms in different modulation modes is calculated. Subsequently, the computational complexity under a typical modulation mode (i.e., 16-quadrature amplitude modulation (16-QAM), quadrature phrase shift keying (QPSK), and binary phrase shift keying (BPSK)), with transmission and receiver antennas as 4 × 4, is figured out and shown in Table 1.

## 5. Simulation Results and Performance Analyses of the Proposed Scheme

### 5.1. Simulation Results and Analysis of Detection Algorithm and Related Sequence Selection

In this section, the detection performance of the proposed scheme and counterpart are simulated under specific conditions. Generally, the experimental verification is the most effective measure to estimate the proposed scheme. However, experimental verification is the same as numerical simulations in nature. It is just as advanced and a true method to verify the proposed algorithm with practical physical parameters and techniques. The effectiveness verification of it is the same as that of the numerical simulations and the latter has good property of much easier implementation and analyses. Therefore, in this section, we mainly use the numerical simulations to verify the effectiveness of the proposed scheme with some theoretical explanation. Compared with the ML algorithm possessing an optimal detection performance currently, the concrete simulation results and analyses are illustrated as follows.

When the signal components are acquired at the receivers of the MIMO-OFDM system, the most suitable signal detection algorithm is first selected to reduce the overall BER of the system. The simulation parameters are set in Table 2 as follows.

**Figure 4:**
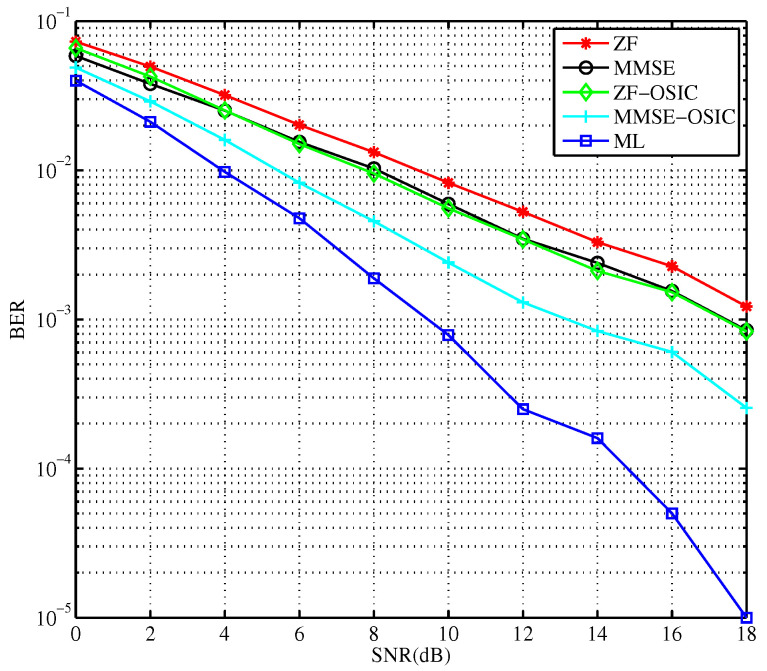
Comparison of detection performance for multiple types of algorithms.

The corresponding detection error rate of each algorithm is obtained as shown in Figure 4. With the increase in the SNR, the detection performance advantage of the optimal ML algorithm become more obvious. However, because of its high computational complexity, it cannot be used in the case of multi-antenna and higher-order modulation.

From the comparison of these detection methods, the optimal detection performance is obtained via the ML detection. But, the computational complexity of the ML detection is huge and it is not suited for the implementation in practice. The detection performance of the MMSE algorithm is better than that of the ZF detection. Combined with the OSIC technique, the proposed MMSE scheme, i.e., the MMSE-OSIC, achieves the sub-optimal performance with acceptable computational complexity for implementation in practice. The performance of the MMSE-OSIC detection obtains a rather good performance, only 4 dB away from that of the optimal ML algorithm for the scheme at BER of $10^{-4}$. The detection performance of the sorted nonlinear detection is better than that of the ordinary nonlinear detection. Therefore, the hybrid detection combining these two algorithms is the better choice to balance both the detection performance and computational complexity together. The following experiment is used to improve the detection performance and reduce the computational complexity of the MMSE-OSIC algorithm. For the MMSE-OSIC algorithm, the detection sequence of detection layers is important. The simulations of the OSIC algorithms via three sorting methods are completed with the simulation parameters in Table 2.

**Figure 5:**
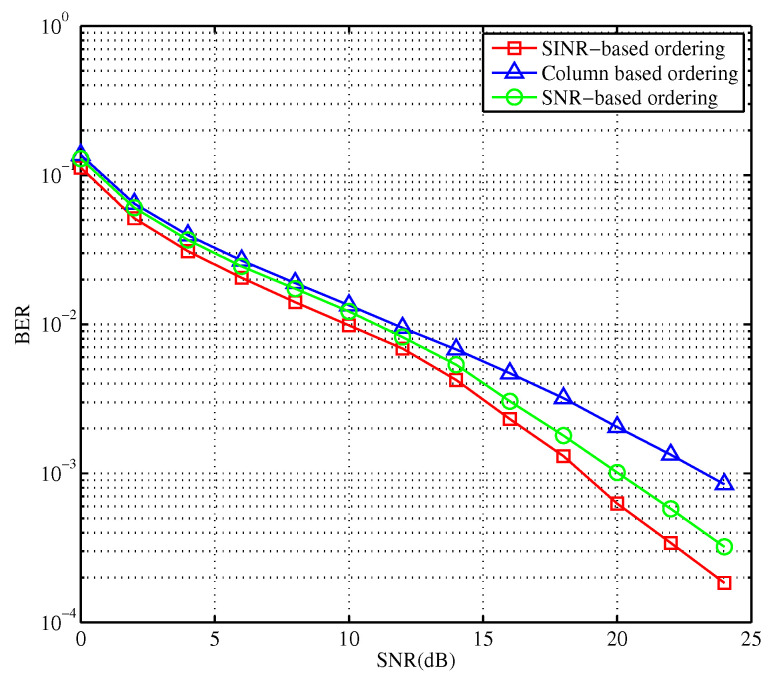
Detection performance comparison of three kinds of OSIC algorithms.

In Figure 5, the performance of the scheme based on the filter matrix column is the worst. Then, the arrangement of the filter matrix is very necessary for the proposed scheme. Compared with the traditional sorting method via SNR, the error performance of the system is improved, when the detection is carried out according to the SINR values with the introduced interference effect. Therefore, the detection scheme and detection sequence of the MIMO-OFDM system are determined. After that, it is necessary to improve the performance and optimize the computational complexity of the proposed algorithm.

### 5.2. Performance Results and Analyses of the System with Improvement

To verify the performance of the simplified algorithm of the matrix inversion, the BER performance and computational complexity of the series expansion and MMSE detections are also compared and analyzed in this section. The simulation parameters are set in Table 3 as follows.

**Figure 6:**
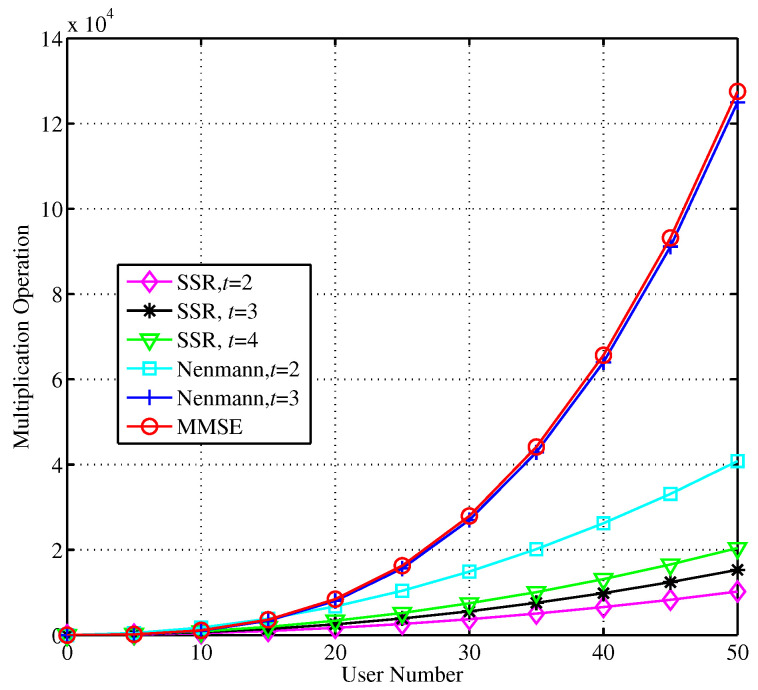
Comparison of computational complexity of different detection algorithms.

The computational complexity via the Neumann series expansion detection, the modified SSOR detection, and MMSE detection is compared in Figure 6. They are mainly measured via a real multiplication operation. *t* represents the number of iterations for the modified SSOR algorithm and expansion terms of the Neumann algorithm.

**Figure 7:**
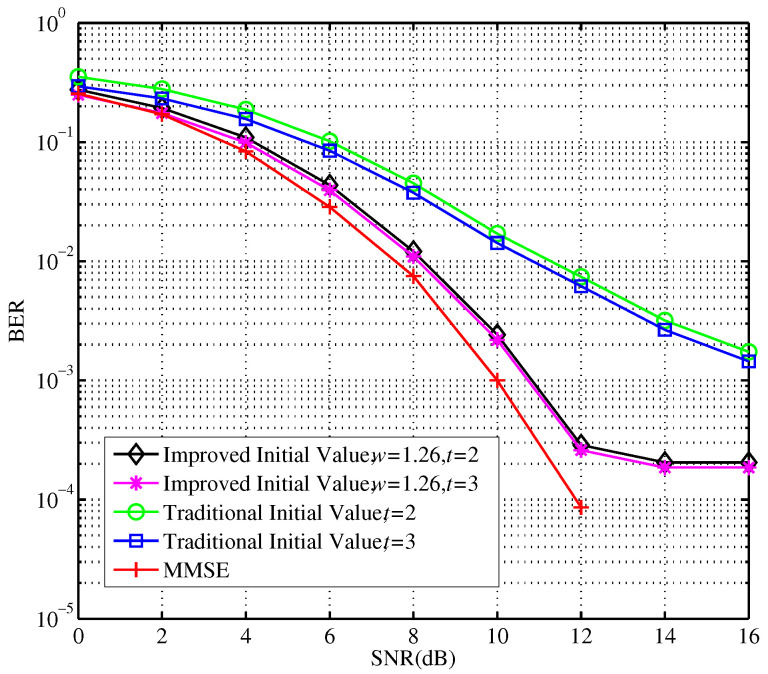
BER Performance comparison before and after the modified SSOR algorithm with improvement.

The experimental results show that the computational complexity becomes higher as the number of iterations increase. When the number of iterations is 3, the computational complexity of the Neumann series expansion scheme is $O(N_{r}^{3})$, similar to that of the MMSE exact matrix inversion process. So, when the number of iterations is greater than 3, the advantage of low complexity is less obvious. By comparison, for any number of iterations, the computational complexity of the modified SSOR iterative approximation algorithm is $O(N_{r}^{2})$. Therefore, the optimum number of iterations are selected according to the detection performance. Figure 7 shows the error performance of the modified SSOR algorithm with different relaxation parameters, iterative initial values, and the number of iterations.

In Figure 7, when the SNR and the number of iterations increased, the performance of signal detection is improved accordingly. Compared with traditional relaxation factors and initial values, the detection performance can be further ameliorated by configuring appropriate quantified relaxation parameters and initial values. Given t=2, the SNR required by the traditional scheme to reach the error rate of $10^{-3}$ is over 16 dB. But, after the proposed improvement, the required SNR is only about 11 dB. Additionally, the speed of convergence is much faster. The choice of the improved initial value can be explained as follows. A modified SSOR algorithm is utilized in an iterative manner to effectively avoid the complex matrix inversion computation. Thus, it significantly reduces the computational complexity of the entire scheme. Additionally. the detailed procedures of the modified SSOR algorithm for this complex matrix inversion computation is given in Section 3.2. From the derivation and analyses of the modified SSOR implementation, especially in (14), the relaxation factor *w* and the iterative initial value must be configured appropriately to obtain better estimation results, or the relaxation factor will have an important influence on the convergence and convergence conditions. For *N*-dimensional linear equations, the relaxation factor must be able to converge the equations to a real solution. To obtain a convergence of (14), the spectral radius of the iterative matrix needs to be satisfied with (16) and (17). Subsequently, a near optimal relaxation factor after quantization is obtained in (19), which is mainly determined by the system parameters $N_{t}$ and $N_{r}$. Therefore, the proposed algorithm can not only significantly reduce the computational complexity, but also needs only 2∼3 iterations to achieve the optimal signal detection.

**Figure 8:**
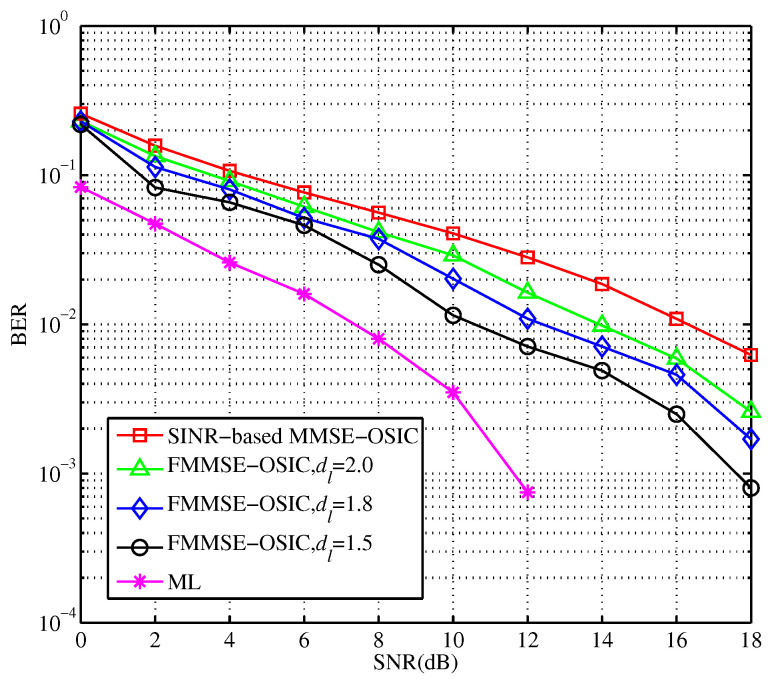
BER performance of the MMSE-OSIC algorithm with feedback under different thresholds.

The performance simulation of the feedback-based MIMO-OFDM system is carried out on different conditions of the antenna number and reliability threshold. The simulation results are shown in Figure 8. Compared with those of the traditional OSIC detection algorithm, when the BER reaches $10^{-2}$, the required SNR of the proposed OSIC detection with feedback is improved about 3 dB at BER of $10^{-2}$. With the increase in transceiver antennas, it is easier to cause error codes in the process of signal transmission. At this time, the feedback output of the algorithm makes the BER performance improvement more obvious. In addition, from the simulation results, the smaller value for $d_{l}$ is, the better the relative decoding performance will be. As the value of $d_{l}$ decreases, the requirements of the decision are becoming tighter. If the number of ML criteria performed by the algorithm increases, then the error performance of the scheme is further improved.

To further improve the system running rate and validate the effectiveness of the proposed OSIC combined with the detection method, the simulation parameters are configured in Table 4 as follows.

**Figure 9:**
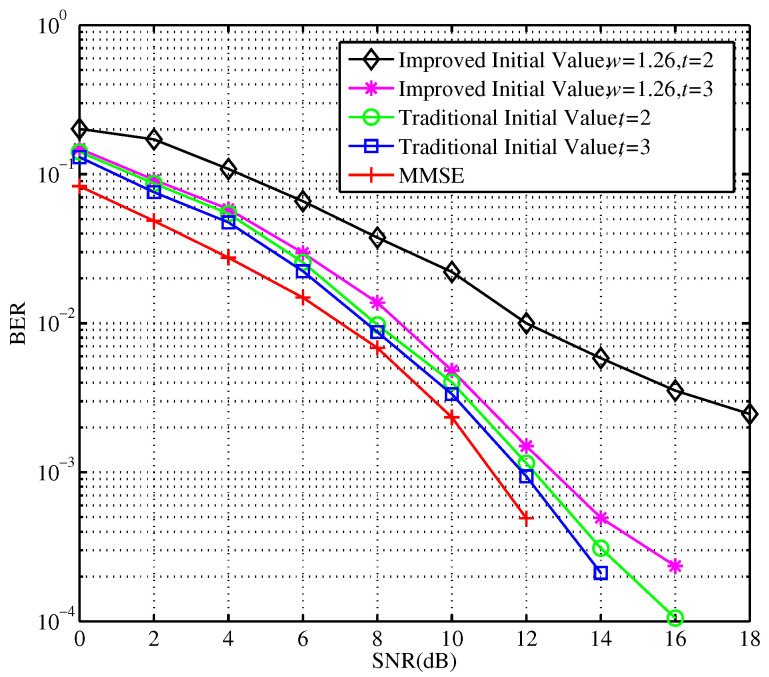
BER performance of m layers joint detection optimization algorithm.

Through the minimum complexity criterion, the first several layers with a better performance of the SINR are selected for joint detection. The simulation results under different parameters are as follows (Figure 9).

From the theoretical analysis, the combined detection can effectively reduce the computational complexity of the OSIC algorithm. When *m* layers are selected for joint detection, the iterative steps of (m−1) layers’ single detection is omitted. From the simulation results, as the number of joint detection layers increases, the detection performance is closer to the optimal maximum Likelihood (ML) detection. Compared with that of the traditional detection layer by layer, the required SNR is improved by about 1dB in the case of same-detection performance. Simultaneously, the improved scheme reduces the running time to about half of the original.

## 6. Conclusions

In this paper, a series of improvements are made for the conventional OSIC detection under the MMSE criteria. An iterative approximation strategy with a quantitative relaxation factor and an iterative initial value is adopted to avoid the matrix inversion process. Compared with traditional relaxation iterative schemes, the convergence speed is further accelerated, while reducing the computational complexity. Simultaneously, by introducing the reliability decision threshold, a further feedback correction is made, which reduces the genetic probability in error propagation and increases the reliability of signal detection at the expense of little extra complexity. Finally, by the minimization of complexity, multilayer joint detection of the improved OSIC algorithm is carried out to reduce a part of the computational complexity. The detection applied in the MIMO-OFDM systems gives consideration to the detection performance and the computational complexity. On the same error rate conditions, the SNR of 4∼5 dB is saved and the computational complexity is reduced significantly from cubic level to square one.

## Figures and Tables

**Table 1:** Computational complexity under different modulation modes (4 × 4).

Algorithm Modulation	16QAM	QPSK	BPSK
ML	3.1 × 10${}^{11}$	4.7 × 10${}^{6}$	1.8 × 10${}^{4}$
ZF-OSIC	700	700	700
MMSE-OSIC b = 1	720	720	720
MMSE-OSIC b = 2	655	581	445
MMSE-OSIC b = 3	475	450	442

**Table 2:** Simulation parameter setting for the selection of optimal detection.

Simulation Parameters	Value
Sub-carrier coefficient	128
Symbol frame length	1000
Modulation mode	QPSK
Antenna number	4 × 4

**Table 3:** Parameter collocation for verifying the performance of the modified SSOR scheme.

Channel Type	Rayleigh Fading Channel
Symbol frame length	25,600
Modulation mode	QPSK
Antenna number	128 × 16

**Table 4:** Simulation parameter collocation for verifying the performance of the improved OSIC schemes.

Channel Type	Rayleigh Fading Channel
Symbol frame length	25,600
Modulation mode	QPSK
Antenna number	4 × 4

## Data Availability

Data are contained within the article.

## Abbreviations

MIMO	multiple-input multiple-output
OFDM	orthogonal frequency division multiplexing
MIMO-OFDM	multiple-input multiple-output-orthogonal frequency division multiplexing
OSIC	ordered successive interference cancellation
ZF	zero forcing
ML	maximum likelihood
MMSE	minimum mean square error
MMSE-OSIC	minimum mean square error-ordered successive interference cancellation
BER	bit error ratio
SNR	signal-to-noise ratio
SINR	signal-to-interference-plus-noise ratio
SSOR	symmetric successive over-relaxation
SLC	square-law combining
16-QAM	16-quadrature amplitude modulation
QPSK	quadrature phrase shift keying
BPSK	binary phrase shift keying
DNN	deep neural network
